# Marital status and educational level associated to obesity in Greek adults: data from the National Epidemiological Survey

**DOI:** 10.1186/1471-2458-10-732

**Published:** 2010-11-26

**Authors:** Themistoklis Tzotzas, George Vlahavas, Sousana K Papadopoulou, Efthymios Kapantais, Daphne Kaklamanou, Maria Hassapidou

**Affiliations:** 1 Hellenic Medical Association for Obesity (HMAO), Athens, Greece; 2 Department of Nutrition and Dietetics, ATEI, Thessaloniki, Greece

## Abstract

**Background:**

Obesity is an important public health issue and its prevalence is reaching epidemic proportions in both developed and developing countries. The aim of the present study was to determine associations of overweight (OW), obesity (OB) and abdominal obesity (AO) with marital status and educational level in Greek adults of both genders based on data from the National Epidemiological Survey on the prevalence of obesity.

**Methods:**

The selection was conducted by stratified sampling through household family members of Greek children attending school during 2003. A total of 17,341 Greek men and women aged from 20 to 70 years participated in the survey and had anthropometric measurements (height, weight, and waist circumference) for the calculation of prevalence of OW, OB and AO. WHO cut-offs were used to define overweight and obesity categories. Waist circumference of more than 102 cm in men and 88 cm in women defined AO. Marital status and educational level were recorded using a specially designed questionnaire and were classified into 4 categories.

**Results:**

The overall prevalence of OB was 22.3% (25.8% in men, 18.4% in women), that of OW 35.2% (41.0% in men, 29.8% in women) and that of AO 26.4% in men and 35.9% in women. Ahigher risk of OB was found in married men (OR: 2.28; 95% CI: 1.85-2.81) and married women (OR: 2.31; 95% CI: 1.73-3.10) than in the respective unmarried ones. Also, a higher risk of AO was found in married men (OR: 3.40; 95% CI: 2.86-4.03) and in married women (OR: 2.40; 95% CI 2.00-2.88) compared to unmarried ones. The risk for being obese was lower among educated women (primary school, OR: 0.76; 95% CI: 0.60-0.96, high school, OR: 0.58; 95% CI: 0.46-0.74 and University, OR: 0.64; 95% CI: 0.49-0.81) than among illiterates. No significant differences were found among men.

**Conclusions:**

In Greek adults, marital status was significantly associated with obesity and abdominal obesity status in both genders while educational level was inversely associated with obesity status only in women.

## Background

Obesity is an important public health issue and its prevalence is reaching epidemic proportions in both developed and developing countries [1]. In Greece, as in most Mediterranean countries, obesity (OB) and abdominal obesity (AO) is constantly increasing in both genders across all ages [2-4].

Body weight is influenced by proximate determinants, which are of genetic and environmental origin, the latter including mainly diet and physical activity. Social characteristics require also special attention because they can have an impact on dietary and physical activity practice. Social characteristics are implicated in the epidemic of obesity and influence body weight by modulating dietary and physical activity patterns. Even if social determinants of obesity are not always modifiable, they may identify and target special subgroups at risk for obesity [5].

Socioeconomic status is frequently indexed using single indicators such as educational attainment or educational level (EL), income, poverty status or occupational status [5]. Unlike income or occupation, educational level is stable, can be assessed for all individuals and is useful across the age spectrum (including among those who are retired or disabled). This indicator does not always reflect the current financial situation of a subject but in most cases reflects its social status. However, EL is subject to reverse causality when examined in association with obesity outcomes. For example, individuals may experience decreases in income, wealth, occupation and increased poverty status with elevated BMI owing to health difficulties, disability and stigmatisation. Lower skill attainment has been shown in obese children compared to normal-weight in some studies, which can affect the educational level they reach [5]. We particularly chose this indicator for our Greek population characterised by patriarchal, traditional views and nuclear families, because it is more "easily and frankly" reported in contrast to other indicators such as income or poverty status. Furthermore the use of occupation could also be misleading because of the high percentage of female unemployment in Greece. In developed societies accumulated evidence suggests an inverse relationship between educational level and BMI particularly among women [6,7].

Marital status (MS) has also been shown to be associated with BMI and most cross-sectional studies tend to find that married people are more often overweight and obese than those living alone; however, important variations exist according to gender and ethnicity [8,9]. It is still not clear how and under what conditions marital status is associated with obesity although interesting hypotheses linking these two outcomes have been raised recently [10].

Only a limited number of cross-sectional studies have examined the relationship of socioeconomic factors, and EL in particular, to obesity in Greece and they were confined to particular geographic areas [11-13]. These studies found an inverse relationship between educational level and obesity status. To the best of our knowledge the association between obesity and marital status in Greek subjects was only marginally examined in one regional study [4].

Understanding the reasons for the prevalence of obesity in Greek adults and determining the socioeconomic factors associated to this condition is very important for the formation of effective public health intervention policies.

The aim of the present study was to evaluate overweight, obesity and abdominal obesity associated to marital status and education level in both genders based on the data analysis of the first epidemiological nationwide survey in adults conducted in Greece [14]. To the best of our knowledge, there has been no study to date that has examined these associations in the whole of the country. Analyses on other factors associated to obesity such as nutritional and physical activity factors will be presented in a forthcoming paper.

## Methods

In this study, data concerning educational level and marital status, coming from the first nationwide cross-sectional epidemiological survey for the prevalence of obesity in Greek adults that was conducted from February to June 2003, were analyzed. The survey has been approved from the ethical committee of the Technological Educational Institute of Thessaloniki (Ref. No 20102). The methodology used for the study was described previously [14]. Briefly, the selection was conducted by proportionate stratified random sampling (SRS) through household family members of Greek adolescents (13-19y) attending public school throughout all parts of Greece. From the 3,514 secondary public schools, a sample of 332 (9.45%) was randomly selected. In each school, according to SRS, all the pupils from four out of six classes participated in the study. Each adolescent received an envelope containing a questionnaire for all relatives in the household. Parental inform consent was provided for the participation of all adolescents in the study. Adolescents were trained by physical training instructor on anthropometrical techniques following standardized criteria in order to take measurements of their relatives at home. Therefore, most adult participants living in the same household were measured for obesity indices by their adolescent children. However this practice has not been validated previously and it is subject to limitations including misreporting of some values. Although a substantial number of Greek adults included in the study were relatives of the adolescents other subjects like young unmarried adults, aged 20-35 (uncles, aunts, older brothers and sisters, housemaids) living in the same house also participated. In the extended Greek family it is common that these unmarried adults live in the same house. Housemaids were also included in the survey. People living in couple without being married were also categorised as "unmarried". However, it has to be mentioned that unmarried couples (cohabitation relationship) with children is not a common situation in Greece.

All participants answered a questionnaire including questions about obesity-associated factors such as nutritional and physical activity habits, educational level, marital status, cardiovascular risk factors, smoking and alcohol consumption habits. The survey was initiated by experienced medical doctors, all members of the Hellenic Medical Association for Obesity (HMAO), with the approval and collaboration of the Greek Ministry of Education. These doctors trained all physical training instructors, who were the responsible for conducting the survey, in selected organized unions in 12 representative cities of Greece. The selection of the population was performed in collaboration with the Department of Statistics of the Athens University of Economics. According to the protocol, measurements for body height, weight and waist and hip circumferences were taken by adolescents of the household. Weight was measured to the nearest 0.1 kg with the same kind of portable scale (Terraillon T 715, Terraillon France, France) and with the participant in minimal clothing. Height was measured to the nearest 0.1 cm without shoes. BMI was calculated as body weight (Kg) divided by squared height (m^2^). WC was measured with a tape at the mid distance between the top of the iliac crest and the bottom of the rib cage. BMI was categorized according to the World Health Organization [15] standards as underweight (< 18.5), normal weight (18.5-24.9 kg/m^2^), overweight (25 to 29.9 kg/m^2^) and obese (> 30 kg/m^2^). Abdominal obesity was defined as a waist circumference of more than 102 cm in men and 88 cm in women [16]. Prevalence of overweight, obesity and abdominal obesity were estimated in the total sample and separately in three-age groups: 20-39.9, 40-63.9 and 64-70 years.

In the questionnaire, educational status was evaluated by asking the participants to declare their highest level of education achievement. Education level was grouped into four categories: a) illiterate or not having finished elementary school b) primary education, c) secondary education and d) university education. Marital status was assessed by asking whether subjects were unmarried, married, divorced or widowed.

From a total estimated number of 22,147 subjects a sample of 17,341 adults entered the study (participation rate 78.3%). The non-response rate (including incorrect values) for the variables included in the analysis was as follows: Body Weight, 2.1%; Height, 2.7%; Waist Circumference, 4.8%; Marital Status, 1.2%; Educational Level, 3.2%. A total of 16,073 participants were available for the final analysis in which 7,579 were men and 8,494 were women. The overall mean age and standard deviation (± SD) was 43.4 ± 19.1 years (for men, 44.4 ± 25.0; for women, 41.3 ± 11.5).

### Statistical Analysis

Multinomial logistic regression analyses were performed to determine the association of gender, age, marital status and education level to overweight, obesity and abdominal obesity. Odd ratios (OR) for overweight and obesity were compared against normal body mass. Age, a continuous variable, was entered as a covariate in the logistic regression analyses that were used to evaluate the effect of marital status and education level on OW, OB and AO for the total population and each gender separately. Interactions between factors entered in the logistic regression models were also examined. Statistical analyses were performed using Minitab v15. Οdd ratios were calculated using a confidence level of 95% and values of p < 0.05 were considered to be statistically significant.

## Results

The mean BMI for the total population studied was 26.4 ± 5.3 and it was higher in men than in women (27.3 ± 4.9 vs. 25.6 ± 5.1). The overall prevalence of OB was 22.3% (25.8% in men, 18.4% in women), that of OW 35.2% (41.0% in men, 29.8% in women) and that of abdominal obesity 26.4% in men and 35.9% in women. Overall sample characteristics are indicated in table 1.

**Table 1:** Overall sample characteristics (n = 16,073)

Attribute		%
Weight status	Normal	42.5
	Overweight	35.2
	Obese	22.3

Gender	Male	47.7
	Female	52.3

Age group (years)	18-39.9	31.3
	40-64.9	63.3
	65-70	5.4

Marital status	Not married	13.4
	Married	80.4
	Divorced	2.7
	Widowed	3.5

Education level	Illiterate	4.3
	Primary school	26.1
	High school	45.6
	University	24.0

Married, divorced and widowed subjects had increased odds for being overweight by 67%, 61% and 94% respectively, compared to unmarried ones (p < 0.001). The respective values for obesity were 62% (p < 0.001), 38% (p < 0.05) and 144% (p < 0.001) while, for abdominal obesity, the values were even higher reaching 114%, 120% and 154% respectively (p < 0.001). (Table 2). Νο 2-way or 3-way interactions among levels of gender, age group, marital status and education level were significant for this first set of logistic regressions (p > 0.05 for all cases).

**Table 2:** Logistic regression analysis for overweight, obesity and abdominal obesity by gender, age group, marital status and educational level

	Overweight		Obesity		Abdominal obesity	
Predictor	Odds Ratio (95%CI)	P	Odds Ratio (95%CI)	P	Odds Ratio (95%CI)	P
**Gender**						
Male	1.00 (Ref.)		1.00 (Ref.)		1.00 (Ref.)	
Female	0.50 (0.46-0.54)	< 0.001	0.72 (0.67-0.78)	< 0.001	1.52 (1.42-1.64)	< 0.001
**Age group**						
18-39	1.00 (Ref.)		1.00 (Ref.)		1.00 (Ref.)	
40-64	1.42 (1.30-1.56)	< 0.001	1.64 (1.48-1.82)	< 0.001	1.57 (1.45-1.71)	< 0.001
> 65	1.52 (1.23-1.88)	< 0.001	2.30 (1.90-2.79)	< 0.001	2.43 (1.99-2.97)	< 0.001
**Marital status**						
Not married	1.00 (Ref.)		1.00 (Ref.)		1.00 (Ref.)	
Married	1.67 (1.45-1.93)	< 0.001	1.62 (1.35-1.94)	< 0.001	2.14 (1.87-2.45)	< 0.001
Divorced	1.61 (1.25-2.08)	< 0.001	1.38 (1.02-1.87)	< 0.05	2.20 (1.73-2.80)	< 0.001
Widowed	1.94 (1.47-2.57)	< 0.001	2.44 (1.85-3.22)	< 0.001	2.54 (1.95-3.31)	< 0.001
**Educational level**						
None	1.00 (Ref.)		1.00 (Ref.)		1.00 (Ref.)	
Primary school	1.02 (0.84-1.25)	0.833	0.83 (0.69-1.00)	0.045	0.97 (0.81-1.17)	0.776
High school	0.94 (0.77-1.15)	0.567	0.76 (0.63-0.91)	0.003	0.98 (0.81-1.17)	0.791
University	0.99 (0.81-1.22)	0.934	0.72 (0.59-0.87)	0.001	0.99 (0.82-1.19)	0.922

The significant association between marital and obesity status was found across both genders, with the exception of widowed men in which the likelihood for being overweight was similar to the one of the unmarried men (Table 3). However, some noticeable gender differences in the overall marriage-obesity status emerged from our study. More specifically, the OR for married men having AO was 3.40 (95% CI: 2.86-4.03), while that of married women was 2.40 (95% CI 2.00-2.88). Moreover, the OR for divorced men being OW was 2.26 (95% CI: 1.41-3.61), for being OB was 2.39 (95% CI: 1.52-3.78) and for having AO it was 3.12 (95% CI: 2.05-4.76), while the respective OR for divorced women were, 1.90 for being OW (95% CI: 1.38-2.62), 1.63 for being OB (95% CI: 1.06-2.49) and 2.38 for having AO (95% CI: 1.77-3.20). Additionally, the OR for widowed women being OW was 3.21 (95% CI: 2.33-4.41), for being OB it was 5.12 (95% CI: 3.60-7.28) and for having AO it was 4.36 (95% CI: 3.24-5.86), while respective OR for widowed men were 1.55 for being OW (95% CI: 0.92-2.59), 2.40 for being OB (95% CI: 1.46-3.95) and 3.53 for having AO (95% CI: 2.21-5.66) (Table 3). No 2-way interactions between levels of marital status and education level were significant for the second set of logistic regressions (p > 0.05 for all cases).

**Table 3:** Logistic regression analysis for overweight, obesity and abdominal obesity by marital status and educational level in both genders (age was entered as a covariate in the model)

		Multinomial regression vs body mass					
		Overweight		Obesity		Abdominal obesity	
Gender	Predictor	Odds Ratio (95%CI)	P	Odds Ratio (95%CI)	P	Odds Ratio (95%CI)	P
**Male**	**Marital status**						
	Not married	1.00 (Ref.)		1.00 (Ref.)		1.00 (Ref.)	
	Married	2.12 (1.80-2.50)	< 0.001	2.28 (1.85-2.81)	< 0.001	3.40 (2.86-4.03)	< 0.001
	Divorced	2.26 (1.41-3.61)	< 0.001	2.39 (1.52-3.78)	< 0.001	3.12 (2.05-4.76)	< 0.001
	Widowed	1.55 (0.92-2.59)	0.098	2.40 (1.46-3.95)	< 0.001	3.53 (2.21-5.66)	< 0.001
	**Education level**						
	None	1.00 (Ref.)		1.00 (Ref.)		1.00 (Ref.)	
	Primary school	0.84 (0.61-1.14)	0.257	0.91 (0.69-1.20)	0.495	0.98 (0.74-1.28)	0.867
	High school	0.77 (0.56-1.04)	0.086	0.83 (0.63-1.09)	0.179	0.98 (0.75-1.28)	0.891
	University	0.85 (0.62-1.16)	0.296	0.79 (0.60-1.05)	0.108	0.97 (0.74-1.27)	0.805

**Female**	**Marital status**						
	Not married	1.00 (Ref.)		1.00 (Ref.)		1.00 (Ref.)	
	Married	2.14 (1.73-2.64)	< 0.001	2.31 (1.73-3.10)	< 0.001	2.40 (2.00-2.88)	< 0.001
	Divorced	1.90 (1.38-2.62)	< 0.001	1.63 (1.06-2.49)	< 0.05	2.38 (1.77-3.20)	< 0.001
	Widowed	3.21 (2.33-4.41)	< 0.001	5.12 (3.60-7.28)	< 0.001	4.36 (3.24-5.86)	< 0.001
	**Education level**						
	None	1.00 (Ref.)		1.00 (Ref.)		1.00 (Ref.)	
	Primary school	1.22 (0.93-1.58)	0.148	0.76 (0.60-0.96)	< 0.05	0.92 (0.72-1.17)	0.497
	High school	1.04 (0.80-1.35)	0.770	0.58 (0.46-0.74)	< 0.001	0.79 (0.63-1.00)	0.051
	University	1.15 (0.88-1.51)	0.296	0.64 (0.49-0.81)	< 0.001	0.92 (0.72-1.18)	0.518

As far as the relationship between EL and obesity is concerned, significant negative correlation was observed between obesity status and EL but not between OW status or AO status and EL (Table 2). Interestingly, the above mentioned correlation was found only in females. Specifically, females with primary school, high school and university education had decreased odds by 24% (p < 0.05), 42% (p < 0.001) and 36% (p < 0.001) to be obese compared to illiterate ones (Table 3). Also it appears that this inverse relationship in women is stronger in high school and university graduates.

## Discussion

This study is the first that reports on marital status and educational level associated to overweight, obesity and abdominal obesity in the adult Greek population. According to our results married, divorced and widowed subjects had higher risk for being overweight, obese or abdominally obese, compared to unmarried ones and this was true for both genders. Concerning educational status, a significant -negative- association between educational level and obesity status was found only in female subjects.

To the best of our knowledge, only one study has examined the association between obesity and marital status in Greek subjects [4]; this was a regional study (ATTICA), which did not find any significant correlations between these two parameters. Most studies that have been conducted in western societies have shown that unmarried individuals are less prone to obesity compared to married ones [17-19], although this relationship may vary by age, gender, ethnicity. Soriguer et al. [20] reported higher obesity prevalence in married subjects up to the age of 45 years, while above this age, divorced persons displayed greatest obesity prevalence. In our study the relationship between obesity and marital status was not dependent on age. In a study by Kilicarslan et al [21], the incidence of obesity was found to be higher in married individuals compared to single or divorced/widowed ones and it was calculated that marriage increased the risk of obesity by 2.5 times. Studies in eastern societies had similar findings as our study in Greece and in other western countries. In a large cohort of 89,404 subjects in Iran, the prevalence of overweight was about twofold higher and that of obesity about threefold higher among married men and women than among never married subjects; the prevalence of abdominal obesity was twofold higher in married men and threefold higher in married women than in non married counterparts [22].

Gender particularities have been described to affect the association between obesity and marital status. In our study, both married men and women were more overweight, obese and abdominally obese than the unmarried ones. However, although divorced men mirrored the married ones in obesity status, divorced women had lower OR for being OW or OB compared to the married ones; an explanation for that could be that separated women re-enter actively the 'marriage market' and pay more attention to nutritional and physical activity issues [10]. Moreover, important discrepancies between the two genders were evident in widows; while widowed men had almost equivalent risk for obesity and AO with married men, much more widowed women than married ones belonged to overweight, obesity and AO status. This could be explained by the fact that, at least in some Mediterranean societies like Greece, widowed women tend to be socially resigned, less focused on being attractive and less physically active. Sobal et al [8] studied in details gender differences in the relationship marriage-obesity status. They reported that compared to married men, white divorced men, black never-married men and more Hispanic men had lower odds of being overweight. Marital status did not appear to have any effect on white women's weights, while black divorced women had higher odds of being overweight, and Hispanic never-married women had lower odds of being overweight. Some gender particularities emerged from the study by Reynolds et al. [23] who compared data from two similar epidemiological surveys in elderly American and Japanese populations. They found that among Americans, marital status had different association with body weight according to gender and showed that married men were 21% more likely to be overweight, while married women were 21% less likely to be obese. In contrast, among Japanese there was no association between marriage and body weight.

The exact mechanism linking obesity and marriage is not fully understood. An interesting longitudinal study in USA examined complete marriage histories in 10,426 subjects from 1979 to 1994 [10]. According to their results, the two most plausible hypotheses explaining BMI increase during marriage are a) the social obligation of marriage, which states that couples are led to eat more regular meals and richer and denser foods and b) the marriage market hypothesis, which suggests that married individuals especially women, who are no longer concerned about attracting a mate may allow their BMI to rise. A third hypothesis, is the selection hypothesis which states that individuals with a lower BMI are more likely to be selected into marriage, and according to the authors, it could also be valuable at least in women.

Educational level is used in many epidemiological studies as a valuable indicator of socioeconomic position. The accumulated evidence suggests an inverse association between EL and obesity mostly among women in developed societies while in men this association is less consistent [19,24-27]. Some studies, however, find this inverse association in both genders [17,20,28,29]. In the bi-ethnic comparative study [23], education level was a predictor of overweight for older Americans but not for older Japanese people. American men and women with higher education had lower possibility to be overweight or obese and each year of education reduced the likelihood by 2-9% [23].

The importance of the role of educational level on obesity epidemic in some populations was also emphasized from prospective studies. These studies evaluated predictors of naturally occurring body weight changes and found EL to be a significant predictor of lower body weight in women [30,31]. AccordingtoArtajelo et al. [26], the rise of overweight and obesity in Spain in the decade 1987-1997 may have been prevented by 1.4% in men and 7.3% in women through the higher educational level.

In our study, when we analysed data in our population as a whole we did not find any significant association between EL and obesity status. However, when we examined associations separately in the two genders, education was found to be inversely associated with obesity status in women. Only few Greek studies of regional origin examined the relationship between EL and prevalence of overweight and obesity. Thus, a previous study in Greek adults of Northern Greece reported higher overweight and obesity prevalence in less educated subjects in both sexes [13]. Similarly, in Southern Greece, obese subjects from both sexes were found to be less educated than non-obese [12].

According to our results, the greatest effect on the development of obesity was observed in women lacking higher educational level e.g. high school and university attainment. This is in agreement with the study by Kilicarslan et al, [21] who found that university graduates were the 62% in the normal-weighing group and only the 31% in the obese group. Noticeably, in a Spanish graduate population EL was independently associated with BMI, even among university graduates; university graduates with a lower EL (college or university degree) of both genders had a significant higher BMI than those with doctorate [32].

Reasons to explain our findings and those of others that the relationship between obesity status and EL is significant only in women arise from the facts that women with a higher EL have better knowledge on issues related to caloric intake and obesity and that social pressures for thinness are probably more intense in educated women than in educated men [31]. The latter fact could also explain the absence of association between AO and EL even in women. Abdominal obesity in women was much more prevalent after the age of 50, and at these ages social pressure for thinness seems less important, at least in Mediterranean societies like Greece. Other explanations for our findings could be that low EL probably increases psychosocial distress, which in turn leads to food overconsumption and decreased physical activity [5].

Some important limitations of the study have to be mentioned. We used only EL as a proxy for socioeconomic position, which, although a reliable indicator in such conditions, it does not always reflect the financial situation of a subject. Another limitation is that anthropometric measurements in our population were performed by adolescents 13-19 y who were trained by school instructors at school. This practice has not been validated previously and errors such as misreporting cannot be excluded; however, all adolescents were carefully instructed to correctly measure and record the anthropometric indices of their relatives. Additionally, the large number of the sample can probably "absorb" some eventual errors, although this is not the case for systematic errors. Besides, the fact that most Greek adults included in the study were relatives of the adolescents could influence results regarding marital status since most subjects belonged to the married category. Ιn fact, no weighting according to national census could have been carried out. In our sample, a cluster effect is also present since several adults belonging to the same household were included in the analysis. Finally, the sampling plan was not taken into account for the process of our data. However, the major strength of the study lies on the fact that this is the first large-scale survey ever conducted in the whole country which provides estimates on the association between obesity, marital status and educational level although in a nonrepresentative manner. Additional positive aspects are the large number of participants and the relatively high response rate.

## Conclusion

In conclusion, data from the Greek epidemiological survey showed that marital status was significantly associated with OW, OB and AO status in both genders while educational level was inversely associated with OB status only in women.

The findings of the present study provide evidence that obesity in Greece, which takes epidemic proportions, could also be faced by tackling specific gender-marital subgroups such as married subjects, which are the majority, divorced men and widowed women. For example, interventions for married couples could include promotion of premarital nutrition education programs in municipalities, promotion of couple physical activities in public gymnasiums, emphasize controlled food portions at the family level and diffuse television and radio programs focusing on healthy eating patterns. For other minority subgroups such as divorced men and widowed women, measures could consist of social support with focus on the counselling of healthy and regular eating patterns, special prices for some foods and physical activity programs etc.

High educational level is also an important issue and it should be promoted along with nutritional education during early life mainly in women.

## Competing interests

The authors declare that they have no competing interests.

## Authors' contributions

TT: participated in the conception and the design of the survey, acquisition analysis, interpretation of data and draft the manuscript. GV: performed statistical analysis and interpretation of data. SP: participated in analysis and acquisition of data and involved in drafting the manuscript. EK: mainly contributed to conception and design of the study and acquisition of data. DK: revised the manuscript critically for important intellectual content. MH: involved in drafting the manuscript and has given final approval of the version to be published. All authors read and approved the final manuscript.

## Pre-publication history

The pre-publication history for this paper can be accessed here:

http://www.biomedcentral.com/1471-2458/10/732/prepub
